# Mixing qualitative methods versus methodologies: A critical reflection on communication and power in inpatient care

**DOI:** 10.1002/capr.12365

**Published:** 2020-11-06

**Authors:** Michelle O'Reilly, Nikki Kiyimba, Alison Drewett

**Affiliations:** ^1^ College of Social Sciences, Arts and Humanities University of Leicester & Leicestershire Partnership NHS Trust Leicester UK; ^2^ Bethlehem Tertiary Institute, School of Social Practice Tauranga New Zealand; ^3^ Faculty of Health Sciences, School of Allied Sciences De Montfort University Leicester UK

**Keywords:** mixed qualitative methods, inpatient care, critical discursive psychology, video‐reflexive ethnography, epistemology, power

## Abstract

This paper offers an illustrative example to demonstrate one way of combining qualitative methods. The context for the study was a UK inpatient psychiatric hospital. Data set one was collected from weekly ward rounds where inpatient staff met with autistic patients to review medication, listen to patient concerns and make plans or adjustments in light of this. Data set two was reflective discursive interviews with patients and staff. The research objective was to critically consider the potential reasons for discrepancies in dissatisfaction reports from patients in the interviews, compared to relative compliance exhibited by patients in the ward rounds. Utilising a video‐reflexive design and critical discursive psychology approach, both data sets were analysed together. It is possible to simultaneously analyse two different data sets, one naturally occurring and one researcher generated because of the epistemological congruence in the overall design. We have presented an argument for the benefits of mixing two qualitative methods, thereby extending the mixed‐methods evidence base beyond the traditional discussions of quantitative and qualitative paradigms.

## 1 INTRODUCTION

The very conceptualising of ‘mixed methods’ typically denotes the combination of a quantitative aspect and qualitative aspect within a study. This type of mixed‐methods approach draws upon two different paradigms and can be referred to as *inter*‐paradigm research. However, another form of mixed methods is to combine two different qualitative approaches, that is *intra*‐paradigm research. Within an overarching qualitative paradigm, different qualitative methodologies or methods can be utilised. This paper aims to demonstrate the value of using mixed qualitative methods by unpacking some of the wider debates in this domain and by illustrating the value of those arguments with empirical examples. To accomplish this, we provide examples of how a mixed qualitative approach is useful to explicate the communication strategies, social practices and epistemic hierarchies inherent in the inpatient environment. The overarching methodology for the study reported in this paper was critical discursive psychology (CDP) and utilised two different data collection methods, the first being naturally occurring recordings of inpatient ward rounds and the second being follow‐up interviews with staff and patients. To address the specific challenges and quality issues that this mixing creates, we first illustrate some of the tensions and arguments that exist in the field.

Most commonly, researchers using mixed methods combine qualitative and quantitative elements within one study, as aforementioned, *inter*‐paradigm projects. The rationale for including two approaches has tended to be to improve the robustness of the research and extend the scope of the findings (Bazeley, 2018). Both quantitative and qualitative approaches have strengths and weaknesses, and by combining both, there is the potential to compensate for any weaknesses in a single research design (Bryman, 2008) and capitalise on the strengths of each approach (O’Cathain & Thomas, 2006). This is important for those working in the field of mental health, as capturing quantitative data can facilitate an understanding of prevalence and provide an overview of the issue, while the qualitative component can provide depth and information about the reasons or contributing factors. Importantly, the quality of the findings for a single‐method or mixed‐method approach depends on the rigour of the process of the research. Thus, when undertaking multiple methods, it is necessary that each component is conducted to the relevant standards and quality markers of that design. One way of promoting this kind of methodological rigour is to work as a research team where members bring with them complementary competencies in different qualitative approaches (Denzin, 2010).

In an inter‐paradigm mixed method study, consideration needs to be given to the relative or perceived value of knowledge gained from its different parts as certain kinds of evidence are positioned as having higher status. In the context of healthcare interventions, the notion of ‘evidence‐based practice’ has become ubiquitous. This concept was introduced by David Eddy, who provided the principles for evidence‐based guidelines (Eddy, 1990). These principles were gradually developed further to encourage more objective decision‐making and to encourage health practitioners to engage with evidence from research (Sackett et al., 1996). Ultimately, these ideas of evidence informing practice translated to a broad spectrum of disciplines, including mental health, education and social care. Despite the diversification of evidence‐based practice across this range of disciplines, the ideology of medical standards has remained rooted in the principles of what constitutes good ‘evidence’. This has led to the argument that quantitative randomised controlled trials (RCTs) are the ‘gold standard’ (Timmermans & Berg, 2003). Randomisation is perceived to be the most effective way of controlling outcome variables to ensure that the intervention is the active agent (Hariton & Locascio, 2018).

This is, however, a position that has received critique, with challenges to the possible undermining of benefits from qualitative approaches (Grossman & Mackenzie, 2005; Lester & O’Reilly, 2015). This is because the expertise and knowledge of practitioners are potentially devalued, and the patient/participant voice is diminished. The consequence of an evidence hierarchy is that those working with qualitative data may feel the need to add a quantitative component to boost the perceived validity of their research or boost its perceived value. However, strong qualitative research has an important role within the wider production of knowledge in its own right. For example, in health settings, qualitative research can inform an understanding of patient perspectives, experiences and treatment needs. We argue that choice of approach and methods should be based on the research problem being addressed rather than an ideology of evidence. Thus, in promoting parity between quantitative and qualitative approaches, it is necessary that the design and implementation of qualitative research comply with the related quality standards of that approach. This has important implications for the mixing of qualitative methodologies and qualitative methods as both components need to be conducted with academic rigour.

The mixing of qualitative methods or methodologies as noted is referred to as *intra*‐paradigm design, as both aspects of the study are drawn from within the same paradigm (O’Reilly & Kiyimba, 2015). In the literature, there are other terms that are used to conceptualise this kind of study, including ‘qualitative mixed methods design’, ‘multiple method design’ (Morse, 2009), ‘multi‐methods’ (Anguera et al., 2018) and ‘combined qualitative methodology’ (Swanson‐Kauffman, 1986). Pluralism is also a term applied to intra‐paradigm mixed‐methods design (Nolas, 2011), but is frequently used to indicate inter‐paradigm mixed‐methods projects (Barker & Pistrang, 2005) as it is a broad concept referring to mixing methods. While there are subtle differences between these conceptualisations, broadly they refer to multiple approaches and/or methods within the qualitative paradigm in one study. Notably, however, this is not necessarily a straightforward endeavour and there is often a misconception that this form of mixing is less problematic than inter‐paradigm mixing (Barbour, 1998). Arguably, this is because the literature has focused on inter‐paradigm debates which have overshadowed more subtle concerns about intra‐paradigm differences (O’Reilly & Kiyimba, 2015). While quantitative research is more homogenous in its epistemological foundation and tends to be underpinned by post‐positivism, within qualitative research there is a greater heterogeneity of epistemological positioning and this is important in the context of these debates.

Notably, it has been suggested that some researchers pay less attention to the impact of mixing potentially epistemologically incompatible qualitative approaches (Wimpenny & Gass, 2000). It may be the case that when combining qualitative approaches, these are poorly anchored within an identifiable epistemological perspective (Caelli et al., 2003). Problematically, when attempting to evaluate or discuss the findings of studies that lack this conceptual foundation, challenges arise in relation to what kinds of claims can be made and how they are presented. In qualitative work, the researcher's worldview influences their epistemic position and should be acknowledged as an important factor in relation to what is regarded as reality (Frost, 2011). Therefore, when designing an intra‐paradigm mixed qualitative study, consideration must be given at an early stage to what the epistemological foundations are of the two proposed qualitative methodologies to evaluate their compatibility (Lambert & Loiselle, 2008). It is necessarily the case that if two approaches arise from the same ontological foundation, there is a greater likelihood of epistemological congruence and greater overall integrity (Annells, 2006).

One of the confusing factors in these debates has been a conflation of the terminology ‘methodology’ and ‘method’. Differentiation between the two is nonetheless crucial, as methodology refers to the overarching approach within which a set of congruent methods for data collection and analysis are embedded (e.g., grounded theory, interpretative phenomenological analysis, discourse analysis are all methodologies). Method refers to the practical details of how data are collected (e.g., focus groups, interviews, naturally occurring recordings) and the process of how data are analysed (e.g., identification of themes, analysis of discourse patterns, generation of theory). Although the common term used is ‘mixed methods’, in some respects, this is a little misleading in relation to combining intra‐paradigm approaches, as in effect this could be mixing two qualitative methodologies.

To address some of the confusion in the field, scholars have worked to differentiate mixed methods from multi‐methods. Unlike mixed methods, multi‐methods are not restricted to inter‐paradigm mixing of quantitative and qualitative approaches (Anguera et al., 2018). Often, multi‐method studies involve multiple types of qualitative research (Tashakkori & Teddlie, 2010).

We take this argument a step further and differentiate between studies that mix two qualitative methodologies and those that mix two qualitative methods. The approach of mixing two qualitative methodologies is referred to as a *synthesised methodologies* study and the approach of having a single methodology with two or more methods of data collection/analysis is referred to as a *mixed qualitative methods* study (O’Reilly & Kiyimba, 2015). Thus, in a synthesised methodologies study, it is important that each methodological component of the study is conducted separately in accordance with the quality parameters of each approach; any integration or ‘mixing’ of the findings occurs at the end. However, with a mixed qualitative methods study using different methods of data collection, the data can be analysed together using a single analytic method and integrated under the rubric of a single methodology, and an overarching methodological design. It is this way of combining methods that we illustrate through our example research and describe in this paper.

## 2 METHOD

Our example for this article is drawn from a communication project on inpatient care. We utilise this example to demonstrate the importance of congruence across decision‐making when undertaking a mixed qualitative methods study. There are several levels that need to be accounted for when conducting this kind of research, and for this study, each of those decisions needed to fit within the critical realist epistemology on which this study was based. In other words, the underpinning world view guiding the study was critical realism, which means that there is an assumed observable reality, the perception of which is influenced by socio‐cultural factors and meaning created through language (O’Mahoney, 2016).

**Figure 1:**
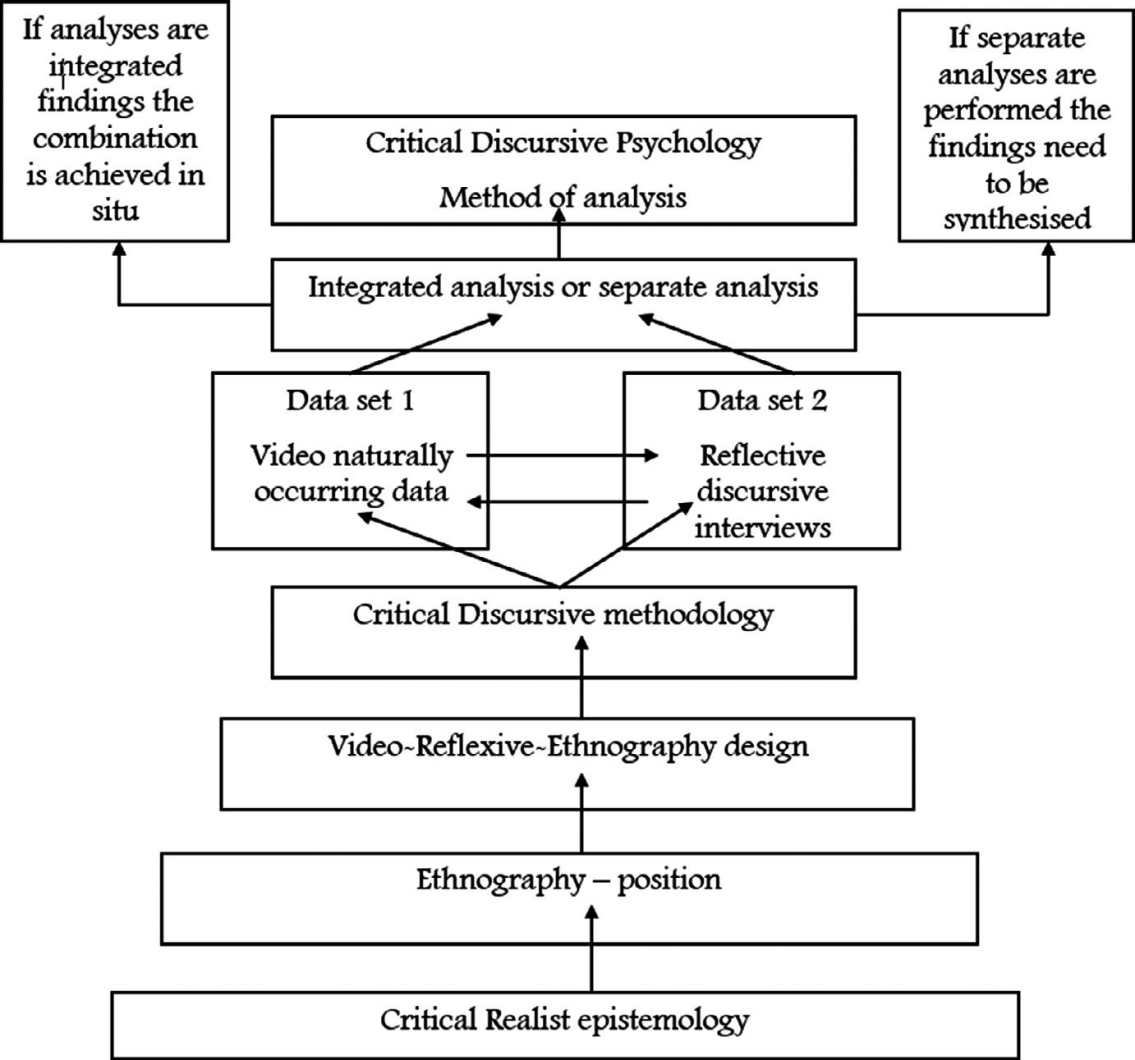
Congruence across design

We employed a video‐reflexive ethnography (VRE) design as a framework to guide the project. The methodology for our work was a critical discursive methodology, which necessitates critical discursive psychology as the analytic method to interrogate the data. The two qualitative methods of data collection were sequentially conducted as dictated by the VRE position taken. First was the collection of naturally occurring video recordings of ward rounds in the unit. Second were reflective ethnographic interviews with a discursive positioning to engage the participants in discussions based on the interactions of those ward rounds. We illustrate this in Figure 1.

### 2.1 Video‐reflexive ethnography

The design of the research was VRE, an approach to investigate how interactional work is accomplished to build change with practitioners through four key principles of exnovation, reflexivity, collaboration and care (Iedema et al., 2019). The essence of this design is data collection via video recordings of real‐world interactions, for example in acute hospital settings (Carroll et al., 2008), palliative care (Collier et al., 2015) and dementia homes (Hung et al., 2018). The priority given to video data to capture the complexity of *work as done* (WAD) rather than *work as imagined* (WAI) (Hollnagel et al., 2015) is used as the focus of conversations with staff choosing VRE as a methodology for applied health studies. The reflexive part connects with the idea that the researcher as insider can use the video data to facilitate reflection and engage with practitioners in the field to review and modify their practices. Often, this is achieved through focus groups or semi‐structured interviews. The ethnographic element of the design is a theoretical alignment with the ideas pertaining to the need for the researcher to understand the field from the perspective of practitioners and to be a part of the setting in which they study.

VRE is not wedded to a specific epistemology or methodology and, as such, authors have highlighted the need for researchers to pay attention to these questions (Carroll & Mesman, 2018). Carroll and Mesman also identify the *clinalyst* as the researcher that capitalises on their insider status, such as the speech therapist's role of the fieldworker here. This is important in health research because this bridges the gap between knowledge generation and knowledge application to practice. We propose that the discursive methodology focus on naturalistic data collection sits comfortably with a VRE design that prioritises video‐recorded data to illuminate tacit practices. Moreover, the critical discursive perspective of the interviews as a social interaction rather than a neutral way to access participants’ inner worlds is also congruent with the principles of VRE. This is because a VRE design does not place constraints on the interviewer to be mindful of reducing biases and maintaining distance with interviewees to extract participant ‘truths’; instead, these conversations are seen as opportunities to share ideas and develop new thinking collaboratively (Iedema & Carroll, 2010). In this research, the interviews were an opportunity to reflect on actual instances from the video recordings that the patient found challenging, and to discuss these findings with staff and patients reflexively. Iedema et al. (2019) also highlight the pervasive presence of power in social interactions, as some practices tend to work to meet the interests of certain people more than others, a perspective that also aligns with the VRE design and with our analytical approach which we now detail below.

### 2.2 Critical discursive methodology

Critical discursive methodology has been defined as the synthesis of a conversation analytic (CA) and a Foucauldian discourse analytic approach (FDA). Styled as a twin approach by Wetherell (1998), a critical discursive psychology (CDP) analysis ‘*pays attention to both the situated and shifting nature of discursive constructions as well as the wider social and institutional frameworks (of meaning of practices of social relations) within which they are produced*’ (Willig, 2013:128). ‘Discourse’ in discursive psychology refers to talk and text as parts of practices, that is ‘*a basic medium of action rather than as an abstract system of description*’ (Potter, 2012:104). In this research example, these were video data from the ward rounds and the interview transcripts. The ‘critical’ approach derives from a lens on such practices as constituted by, and constitutive of, wider power relationships both within and outside of the setting under the gaze of the researcher.

### 2.3 Context and setting

The setting for the research was a UK psychiatric hospital containing separate units for acute, intensive and rehabilitation services. The recordings covered all three areas. The adult patients include three male and three female autistic individuals all with comorbid diagnosed mental health difficulties, and all had been inpatients for a period of more than six months. We recognise that there are differences of opinion regarding the use of either person‐first (person with autism) or condition‐first (autistic person) language in the context of autism. However, through our paper, we elect to utilise condition‐first language, as evidence illustrates this is the preference for most of those with an autism diagnosis (Kenny et al., 2016). Ward rounds are staff and patient discussions held in meeting rooms where families can also attend, which typically take place weekly to review patient progress and care management.

### 2.4 Data collection methods

The mixing aspect of this study was at the level of methods and *not* methodology. Thus, we utilised two methods of data collection which were sequentially undertaken as consistent with a VRE design, whereby one method enabled a reflection on the other in situ. The first method of data collection was to video record naturally occurring ward rounds with adult autistic patients and staff. We recorded six of these ward rounds, which ranged from 12 min to 38 min, with a mean of 25 min. Naturally occurring data are especially useful for healthcare research and refer to recordings of naturally occurring events without interference from a researcher (Kiyimba et al., 2019). This means that the event or situation would occur naturally, even if the researcher were not able to attend to record it (Potter, 2002). Although arguably this cannot be fully ‘natural’ as the presence of the recording device is ever‐present due to ethical parameters, evidence shows that participants become quickly acclimatised to its presence and it has little or no effect on the interaction (Speer & Hutchby, 2003).

We then conducted separate reflective ethnographic interviews in a discursive style with members of the ward round, including the autistic individual and two of the members of staff. We thus collected data from 18 individuals from ward rounds and analysed the communication processes and content through this data collection method. Reflective ethnographic interviews are designed to explore the meanings that the individuals ascribe to aspects of their lives and to explore in depth their cultural worlds (Roulston, 2010), and to be consistent with our methodology, we conducted these through a discursive lens to enable socio‐political structures to be identified.

### 2.5 Analysis

Congruent with our overarching design, positionality and methodology, we conducted a CDP analysis of both the video data and the interview data. CDP has a broader focus on patterns within the talk rather than sequences of talk as in CA (Wiggins, 2017). Scholarship within the CDP tradition has focused on three analytical concepts: interpretive repertoires or the ‘*stock of shared cultural understandings*’ (Horton‐Salway & Davies, 2018:17); ideological dilemmas, for example arising out of apparent discordance between ways of talking about particular topics (Billig et al., 1988); and subject positions relating to ways of representing what is possible within role identities. CDP ‘*seeks to identify the ways in which people are positioned in particular ways and how repertoires are reproduced and held to be common‐sense*’ (Horton‐Salway & Davies, 2018:46). In brief, it marries the discursive psychology (DP) focus on how individuals use discursive resources and the Foucauldian discourse analytic (FDA) interest in what subject positions are made available (Willig, 2013). It is also congruent with the use of naturalistic data and interview data, unlike stricter DP and CA methodologies. It is suited to VRE as it allows the context of the data collection to be an analytical feature so that setting can be examined, as well as participant orientations.

### 2.6 Ethics

The research gained ethical approval from the Health Research Authority following a robust and in‐depth process. Initial concerns regarding the vulnerability of the patients because of their medically diagnosed mental health condition and (formal) inpatient status, as well as the video data collection method, were resolved satisfactorily before data collection commenced. For participant care, all access to patients had to be conducted via an initial introduction from a member of the patient's clinical team. Pseudonyms were allocated to all participants at the point of transcription and are used throughout this paper. The ethical protections put in place benefitted from the insider status of the main researcher, especially her clinical experience of working with autistic adults, her knowledge of safeguarding processes and of hospital procedures. In this sense, reflexivity underpinned the research in a foundational way.

### 2.7 Methodological reflection

The team of researchers working on this project consisted of an academic chartered health psychologist and sociologist (MO), a clinical‐academic chartered clinical psychologist (NK) and a clinical‐academic speech and language therapist (SALT) (AD). Two of the team had professional clinical experience of working in inpatient psychiatric units in the UK, and the SALT had experience of working in the specific inpatient unit where the data were collected. There are two ways in which this type of team composition benefits research evidence. First is the partnership working between clinicians and academics which facilitates the application of theory to practice and allows for practice experience to inform development of theory. Second is the combination of insider (emic) and outsider (etic) knowledge and experience, which has the benefit of enabling the research team to make sense of the ways in which participants explain their understanding of their experiences. It is acknowledged that there are significant strengths to each of these positions, but also the potential for blind spots or biases. For example, from an ethnographic perspective, insiders may develop more sympathy for participants in their study which may influence the ways in which they conduct their analysis (see Hammersley & Atkinson, 2010). Conversely, outsiders may miss the nuances or importance of certain contextual information (see Bosk, 2003). By working together, open dialogue and sharing ideas facilitate reflection on these potential dynamics to promote transparency and maximise integrity in the way data are analysed and reported.

## 3 ANALYSIS

**Figure 2:**
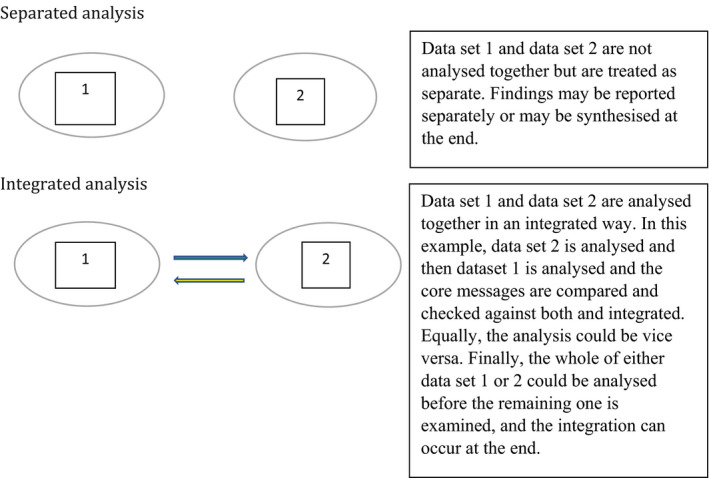
Illustrating integration and separation

With two compatible data sets, there are two different ways of approaching the analysis. In Figure 1, we demonstrated that this would be either through a separated or integrated analytic process. First, a separate approach would be to analyse both data sets independently using inductive enquiry without reference to the other data set. This would produce two separate analyses which could then be synthesised to provide an overarching combination of findings to answer the research question. Second, an integrated approach would be to analyse both data sets simultaneously using a mixture of inductive and deductive enquiry. This involves moving dynamically between the two data sets. This could mean that data set one is analysed inductively, and when key analytic messages are identified, the researcher actively seeks comparable examples in data set two using a deductive approach. Alternatively, data set two could be analysed first through inductive enquiry and data set one interrogated deductively using lessons learned from data set one. This is an iterative and dynamic process where movement between two data sets can occur throughout the analysis. See Figure 2.

It is this latter integration approach to analysis that we illustrate through our examples. In this case, data set two, the interviews were analysed first using the inductive approach of CDP. CDP is underpinned by a critical realist epistemology that assumes a real and observable world containing dynamic features such as power, inequality and oppression. One reason for utilising this methodological approach was to be able to explore these power dynamics within the inpatient setting. The core issues at stake from the perspective of the patients in data set two formed the basis of mapping analytic findings onto data set one. In other words, when examples of these power dynamics were identified in data set two, data set one was then scrutinised to further explore what the patient ward round revealed about what had been reported in the interview. Thus, a more deductive approach was taken to analysing the ward round data. Using this approach, patient reflections in their interviews could be directly compared with the interactions that occurred within the ward round that they referred to. While it would be plausible to examine data set one first and subsequently identify domains of interest in data set two, we started with the reflections within the patient voices first. By simultaneously examining these two data sets, a more holistic understanding of patient experiences could be ascertained.

We provide two illustrative examples of the process of integrating analysis and combining core findings. Our first example illustrates power operating in the inpatient care setting in the context of a medication adherence discussion, and our second example illustrates power through dialogue around the anxiety invoked in trying to communicate with a professional who is perceived by the patient as unwilling to listen to her views and lacking empathy. Both examples highlight the challenge of empowering patients and genuinely hearing their views within the inpatient context. We acknowledge here that ordinarily CDP would have a lot more depth. However, as we are utilising this analytic approach for illustrative purposes only, we simply highlight a few key points and observations to demonstrate the value of the mixed qualitative methods analytic approach (and to contrast with the synthesised methodologies approach). This is not intended to be a full CDP analysis of the data.

### 3.1 Example one—medication

A long‐standing discourse with the medical profession is around adherence to prescribed medication consumption. From the medical perspective, it is important to ensure that patients consistently take the medication they have been prescribed; however, it is a well‐known challenge that patients often do not consistently comply with doctors’ recommendations (Velligan et al., 2017). This dynamic is illustrated in the first example from the patient's perspective. In data example one from the ward round, the psychiatrist strongly advises the patient to comply with the medication strategy. In data example two from the patient interview, the patient explains the reasons why they would prefer not to take this medication. The mismatch between the outcome from the ward round and the explanation from the patient interview indicates that one possible reason for non‐compliance could be a rupture in the way that conversations about medication options were discussed and agreed upon in the ward round.

Through an interrogation of the interview with Tom, we identified an example of the patient illustrating something as problematic in the ward round. The problem identified by Tom was a concern about the negative side effects of taking medication for his psychotic symptoms, and his difficulty in communicating those concerns to medical team.

Interview data—From Tom.

Tom: Um…not so good because my main concern is the medication and I feel like they're not really listening or taking on board what I’m saying or what I want. Because I didn't want to take it in the first place. And I feel like it's having a negative effect on my mood and my mental health and wellbeing.

…

Ali: Mmm. Have you had an opportunity to talk to anybody about that?

Tom: Yeah, I’ve spoken to Dr Archer but she thinks it's best that I should take it anyway.

Ali: Okay, and has she explained to you why she thinks it's best?

Tom: Um, she has but I don't think it's worth it. To me it's not worth it because it's injuring my life, my wellbeing.

Ali: Mmm, and is it helping with the psychotic symptoms at all?

Tom: Not to me, no.

The extract opens with Tom expressing his ‘*main concern’* as positioning the inpatient unit staff as not currently ‘*listening*’ or responding to his worries regarding his prescribed medication and its side effects. In so doing, he positioned himself as someone who had insight into the impact of the medication on his ‘*mood, mental health and wellbeing*’ but having that epistemic right ignored or treated as unimportant. Although Tom reported that he had the opportunity to express his concerns to the psychiatrist, her decision remained that it was in his best interest to continue taking the medication. Interestingly, the same sentence construction formulation was employed by Tom (but she thinks; but I don't think) that the doctor had the chance to explain her rationale, but he disagreed with this. The contrast between the doctor's version ‘*she thinks its best*’ with Tom's version ‘*I don't think it's worth it’* are marked by disclaimers (Potter, 1996) to illustrate the disagreement. In some respects, an ideological dilemma (Billig et al., 1988) is presented between the patient's sense of knowing what is best for him versus the dominant medical discourse of the doctor knowing what is in his best interest. Given that the patient was engaging in the social action of complaining during the interview about decisions that were made during the ward round about medication options, it was valuable to explore the corresponding naturally occurring event being discussed.

Ward round data—from Tom

Psych: We can try it ((Aripiprazole)) and if you take it in tablet form, we'll have a good idea in a few weeks as to whether it works well for you or not, or whether you're having any side effects to it.

Tom: I don't want to take it really.

Psych: You don't want to take the tablet?

Tom: No.

Psych: In which case we would need to carry on with the injection.

Tom: Yeah.

This extract follows a discussion about the relative benefits and side effects of the Clopixol depot injection versus Aripiprazole in tablet form. In concluding that discussion, the psychiatrist suggested that Tom could stop taking the injection and instead begin to take a different antipsychotic in oral tablet form. At this point, Tom asserted ‘*I don't want to take it really’*. The psychiatrist picked up on the ambiguity of the indexical referent ‘it’ from Tom's turn and clarified whether it was the tablet that he did not want to take; a proposition that achieved confirmation ‘*no*’. What becomes clear at this point is that the conversation is about either taking the injection or the tablet, but there is no option of not taking medication. This is evident in the statement made by the psychiatrist that if Tom did not take the tablet then he would need to ‘*carry on with the injection’*. It is simply one or the other.

The overarching interpretive repertoire evident across the interview and ward round is the notion that those individuals labelled as experiencing ‘psychotic symptoms’ ought to be treated through medical intervention from the discipline of psychiatry. Thus, through the abnormalisation and medicalisation of the experience of hearing voices and seeing spirits, an interpretation is made about what the appropriate ‘treatment’ would be to facilitate normalisation. Wellness, in this medical framing, is defined as the absence of symptoms or experiences that are outside of the parameters of cultural norms, and medical professionals are in positions of power as gatekeepers to enforce compliance. Importantly, psychiatrists are working within a specific medical paradigm that operates on the premise of the patient's best interests within that cultural repertoire. Within this repertoire, psychiatrists have a limited amount of flexibility about which medications can be prescribed that are licensed for particular illnesses. At the same time, in keeping with mental health recommendations from professional regulatory bodies regarding patient choice, shared decision‐making and patient‐centred care, psychiatrists are expected to engage patients in the processes related to their treatment. In reality, this means discussing patient preferences between one medication and another. While this does offer some choice, there is no option to not take medication. The asymmetry across subject positions can be further explored as we present example two.

### 3.2 Example two—anxiety

Most (but not all) patients in psychiatric inpatient settings are in these environments because of the application of a mental health section, which has been put in place to restrict their liberty to keep them and/or others safe. In addition to their underlying mental health condition, patients are often highly anxious about ward rounds because these are the times during the week whereby important decisions are made about how long they are likely to have to stay, what kind of treatment and medication they need and what, if any, rights to community leave they will be allowed to utilise. In this example, analysis from data set two revealed that the patient had experienced anxiety about attending the ward round due to anticipated concerns that the psychiatrist would not be empathic or listen properly to her point of view. With this knowledge ascertained from data set one, the ward round data were explored to understand what had been said or done that might lead the patient to feel that way.

Interview data—from Pip

Pip: from my point of view I don't get any kind of…like comfort that I’ve been understood or heard in any way……

*Lines omitted*

Pip: I’ve been really anxious about ward rounds because I just don't want to have the stress of talking to him. Because I just know it's going to be stressful even before I’ve gone in. It's…yeah.

Ali: Yeah…and…it was apparent to me that he didn't say very much.

Pip: Yeah, he doesn't. That wasn't unusual, although the ward round you were in… So, I know you were going to be there, but I was expecting you, the doctor, a nurse and maybe a couple of junior doctors. And then I walked in and there were like 14 people in the room.

Like Tom's interview, Pip revealed here that she also did not feel ‘*understood or heard*’ by the medical team in charge of her care. Her formulation was quite extreme and seems to express dissatisfaction with the way in which the ward round progressed. Indeed, she described the event as ‘*stressful*’ and, specifically, communicating with the psychiatrist was positioned as causing her ‘*stress*’. The asymmetry between the patient’s anxiety (reported to be caused by the ward round) was contrasted with a repertoire of medical institutional business as Pip used medical subject positions of ‘*doctor*’, ‘*nurse*’ and ‘*junior doctor’*. This was extended, however, through the inclusion of a specific number of medical professionals, ‘*14 people*’, to illustrate the differential of one patient in an environment crowded with medical staff. This significant numerical difference was constructed by Pip as ‘*wasn't unusual*’ to indicate that there were not usually quite that many professionals present at her ward rounds. Like our analytic process with extract one, this expression of the identity of the patient within the ward round event, and the description of the anxiety created by attendance prompted us to look on the ward round itself to explore how this anxiety was expressed in situ.

Ward round from Pip

Dad: I think it's the…Pip's perception was that the person thought that Pip had more control over it than Pip feels she has. Um, and the lack of control and the lack of memory of what goes on during these periods is really scary for Pip. And so, um, and she's been struggling to understand that, let alone control that I think over the last number of weeks. And we've raised it [raised hand gesture with slicing hand movement] in the last few ward rounds as well [hand movement in air and circled], as you know, yeah.

*Lines omitted*

Psy: Can I just clarify, do you mean that it is not related to the timing of the medication?

Pip: No, it is.

Psy: It is? So, is it happening before you get your medication?

Pip: Yeah…yeah. When the medication starts to wear off, that's when it happens.

Psy: Okay. Do you think it could disappear if you take more medication?

Pip: I don't…no, I don't think—I don't know. I don't know if you understood me.

In the context of the example we present here, Pip's father was present in the ward round and provided an additional voice to represent and advocate for the patient. We open this example where the father presented a version of events to the psychiatrist in response to his daughter becoming visibly distressed during the questions from the psychiatrist. The discussion focused on episodes of dissociation and self‐harm, which were reported to be considerably ‘*scary for Pip’*.

The discussion about alleviating the symptoms of headbanging and the associated distress related to the timing of when this had been happening, coinciding with the medication starting to ‘*wear off’*. Understandably then, the psychiatrist offered the medication‐related solutions of either increasing the dosage or changing the timing of administration. However, what these potential solutions miss are that Pip had previously expressed that she wanted to understand why she head bangs and why she cannot remember, and more broadly wants to find ways to improve her mood. In short, her agenda was more explicitly about patient education, while the team's priority was related to preventing risky behaviours by medicating. This impasse, or mismatch between staff and Pip's aims, meant that Pip continued to feel not listened to and frustrated.

## 4 DISCUSSION

While traditionally mixed‐methods scholars have attended to the polemic of inter‐paradigm combinations, in our paper we have illustrated a more nuanced argument regarding the intra‐paradigm benefits and challenges. By focusing specifically on the differentiation between mixing qualitative methodologies and qualitative methods, we have provided a solution‐focused way of attending to the epistemological debates. In this article, we have utilised a larger study of inpatient communication to illustrate some of the technical and pragmatic ways of engaging in a qualitative mixed‐methods study. In so doing, we have shown how two different qualitative data sets can be complementary, and can reveal different facets of the social actions and subject positions of staff and patients within an inpatient psychiatric environment. Having these two complementary perspectives enables a deeper and richer, as well as a more comprehensive, insight into communicative practices, processes and dynamics of care.

In this specific research project, it was anecdotally observed that there was a discrepancy between decisions made in ward rounds and patients’ reported satisfaction with those decisions in conversations outside of them. Thus, it was agreed that it was necessary to access the discussions that occurred in both contexts to truly understand the (mis)communication dynamics in the inpatient environment. To accomplish this, a research design that anchored two domains of interest together was necessary to ensure congruence both theoretically and pragmatically, and thus, a VRE design was chosen as the most appropriate way to do this. By utilising a mixed qualitative methods study, whereby two methods of data collection were combined, as opposed to a synthesised methodologies study, it allowed for an integration of data analysis using a single analytic approach. By utilising two forms of data collection, the researchers were able to explore specific issues in more depth. For example, the interviews illustrated that patients were not entirely satisfied with decisions made during the ward rounds, but by examining the naturally occurring recordings from real ward rounds in detail, compliance and adherence were evident. Thus, potentially due to the power dynamic, it seems that patients were complying with decisions in situ that they were not entirely happy with. It is only by combining the interview data with the real world naturally occurring data that these discrepancies could be identified. If only one or the other data set were available to an analyst, a skewed understanding would result. New forms of knowledge and understanding of power in mental health settings are therefore made possible by the evolution that has occurred within the qualitative paradigm that has seen a shift through methodological moments towards critical ideas concerning socio‐political constructs (Denzin & Lincoln, 2018). As Denzin articulated, ‘*moral and epistemological discourses now go on side by side’* (2010, p. 424).

When undertaking mixed qualitative methods studies, the composition of the research team is important. For our project, we carefully addressed the value of working in partnership by planning the research team design. Our priority was to ensure a combination of emic (insider) and etic (outsider) contributions to achieve a holistic understanding of the way participants make sense of their experiences. The utilisation of an emic approach is especially useful when collecting and analysing data, and the etic approach facilitates the application of concepts and theories in the interpretation of findings (McCann et al., 2017). The research team for this project comprised academic and clinical‐academic experts, which meant that different kinds of complementary knowledge could be drawn upon. The experiential emic approach juxtaposed with the objective analytic knowledge of the etic approach, when combined helps to ensure that the interpretation and analysis of data are robust and credible.

Fundamentally, the inter‐paradigm debates have largely arisen due to the challenges of the lack of epistemological congruence across quantitative and qualitative methods. However, what we have illustrated in this paper is that a mixed qualitative methods study where two methods of data collection are integrated under a single methodological approach has epistemological congruence. Where two methodologies are combined, they are not mixed, rather they are synthesised after the quality indicators for both separate approaches have been adhered to. In this approach, any ‘mixing’ cannot occur until both aspects of the study are complete. In conclusion, the decision to undertake a qualitative mixed‐methods study should not be informed by a fear that a single study is insufficient; rather, there should be a clearly identified aspect of added value determined by combining two methods of data collection and integrating (or separating) the analysis derived from them.

## DISCLAIMER

The study from which the data are drawn is funded by the National Institute for Health Research (NIHR) Applied Research Collaboration East Midlands (ARC EM). The views expressed are those of the author(s) and not necessarily those of the NIHR or the Department of Health and Social Care.
